# Acidic pH Promotes Refolding and Macroscopic Assembly of Amyloid β (16–22) Peptides at the Air–Water Interface

**DOI:** 10.1021/acs.jpclett.2c01171

**Published:** 2022-07-15

**Authors:** Hao Lu, Luca Bellucci, Shumei Sun, Daizong Qi, Marta Rosa, Rüdiger Berger, Stefano Corni, Mischa Bonn

**Affiliations:** † Max Planck Institute for Polymer Research, Ackermannweg 10, 55128 Mainz, Germany; ‡ NEST − Istituto di Nanoscienze del Consiglio Nazionale delle Ricerche CNR-NANO and Scuola Normale Superiore, Piazza S. Silvestro 12, Pisa, 56127, Italy; § Department of Physics and Applied Optics Beijing Area Major Laboratory, Beijing Normal University, Beijing, 100875, China; ∥ Istituto di Nanoscienze del Consiglio Nazionale delle Ricerche CNR-NANO, 41125 Modena, Italy; ⊥ Dipartimento di Scienze Chimiche, Università di Padova, 35131 Padova, Italy

## Abstract

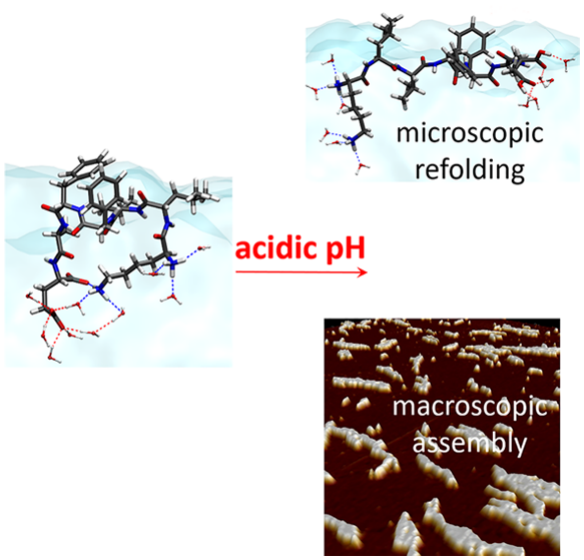

Assembly by amyloid-beta (Aβ) peptides is vital for various neurodegenerative diseases. The process can be accelerated by hydrophobic interfaces such as the cell membrane interface and the air–water interface. Elucidating the assembly mechanism for Aβ peptides at hydrophobic interface requires knowledge of the microscopic structure of interfacial peptides. Here we combine scanning force microscopy, sum-frequency generation spectroscopy, and metadynamics simulations to probe the structure of the central fragment of Aβ peptides at the air–water interface. We find that the structure of interfacial peptides depends on pH: at neutral pH, the peptides adopt a less folded, bending motif by forming intra-hydrogen bonds; at acidic pH, the peptides refold into extended β-strand fibril conformation, which further promotes their macroscopic assembly. The conformational transition of interfacial peptides is driven by the reduced hydrogen bonds, both with water and within peptides, resulting from the protonation of acidic glutamic acid side chains.

Misfolding of proteins and peptides into amyloid fibers is the hallmark of more than 50 human disorders, including Parkinson’s, Alzheimer’s, and prion disease.^1^ These neurodegenerative diseases are primarily caused by the deposition of amyloid plaques composed of amyloid-β (Aβ) peptide fibers.^2^ Because of the great health relevance, considerable efforts have been made to improve our understanding of the assembly and fibrillation of Aβ peptides.^3−5^

Aβ peptides consist of 40–43 amino acids, its central domain is the 7-amino acid sequence from residue 16 to 22, i.e. “KLVFFAE” abbreviated as Aβ_16–22_.^6^ As shown in Scheme 1, the Aβ_16–22_ peptide has the hydrophobic core (LVFFA) and hydrophilic terminals (K and E). This amphiphilic sequence has been widely used as a model system for investigating the assembly and fibrillation of Aβ peptides.^7−9^ In aqueous solution, the Aβ_16–22_ peptides lack a defined conformation, but tend to assemble into β-sheet fibers. The fibrilization process can be promoted by increasing hydrophobic contacts.^10^ In addition, the assembly and fibrillation can be affected by varying the peptide charge and/or modifying the chemical capping of terminal amino acids.^7^

**Scheme 1:**
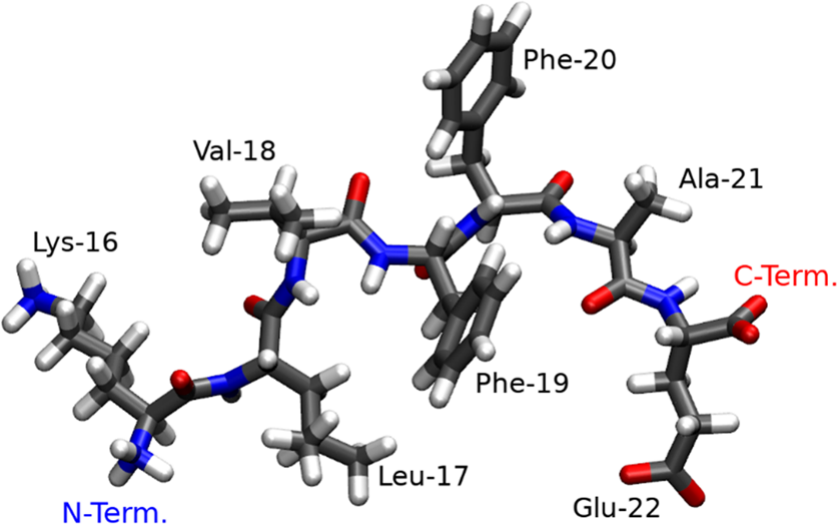
Molecular Structure of the Aβ_16–22_ Peptide At neutral pH: LYS and the N-terminal group are protonated and positively charged, while the GLU and C-terminal are deprotonated and negatively charged. Under acidic conditions the carboxyl group of GLU and that of the C-terminus are neutral as they are both protonated.

Available studies on the assembly of Aβ peptides focus on bulk solutions, while these studies also emphasize the importance of hydrophobic interfaces, such as the cell–membrane^5,11^ or air–water interface,^12^ which can significantly catalyze the fibril assembly for amyloid peptides. Understanding the role of the air–water interface for peptide assembly is also important for *in vitro* studies of amyloid proteins and peptides, given the ubiquity of that interface in such studies. Finally, the investigation of the air–water interface allows pinpointing the specific role that extended hydrophobic interfaces have in promoting assembly, disentangling it from other contributions (e.g., charges, specific interactions) present for, e.g., a cell membrane. Therefore, obtaining molecular level insights of Aβ_16–22_ peptides on such model interface is important step before studying their behavior at complex lipid interface.

Recent simulation studies have found that zwitterionic Aβ_16–22_ peptides adopt major β-turn secondary structure at the air–water interface,^9,13^ while experimental proof for the structure of interfacial peptides is rather lacking. The charge of Aβ_16–22_ peptides can be conveniently modified by solution pH, while molecular response of interfacial peptides on pH is still unknown, one better understanding would provide needed insights into the driving force for their interfacial assembly. For this, we investigate the Aβ_16–22_ peptides at air–water interface with varying solution pH. We find acidic pH triggers the refolding of interfacial peptides into β-strand fiber conformation, which promotes their macroscopic assembly.

In a first experiment, we transferred the Aβ_16–22_ peptides assembled at the water surface at pH of 7 and 3 onto atomically flat mica substrates (details of sample preparation in the Supporting Information), and examined the surface by Scanning Force Microscopy (SFM). The interfacial peptides at pH 7 show no specific feature (Figure 1a). However, the interfacial peptides at pH 3 assemble into elongated flakes, which are evenly distributed on the mica surface (Figure 1b). The flakes have length up to 350 nm and a diameter of 10–60 nm. We analyzed the height of more than 10 flakes and obtained a mean height around 2.6–3.4 nm, and the values agree with the dimension of the interfacial peptide molecule.

**Figure 1:**
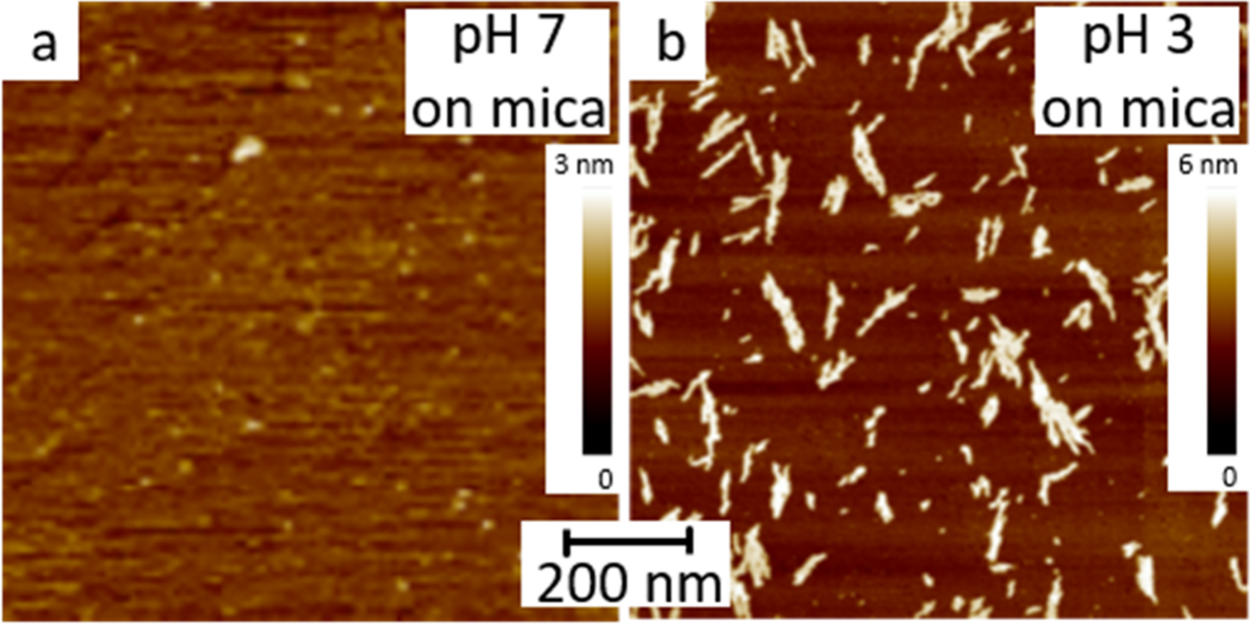
(a, b) SFM images of the transferred films of Aβ_16–22_ peptides at air–water interface with a bulk solution pH of 7 (a) and 3 (b).

To better understand the macroscopic assembly of interfacial peptides, we probe their microscopic structure using interface-selective sum-frequency generation (SFG) spectroscopy. SFG spectroscopy is a nonlinear optical technique relying on the frequency mixing of infrared and visible laser pulses. Molecular resonances excited by the infrared pulse enhance the signal, yielding a vibrational spectrum of interfacial molecules. The SFG selection rules dictate that the signal can be generated only from ordered interfacial molecules where centrosymmetry is broken,^14,15^ this renders SFG spectroscopy an ideal tool to probe the structure of the Aβ_16–22_ peptides specifically at the air–water interface.^16−18^

Figure 2a shows the SSP SFG spectra in the frequency region of 1440–1780 cm^–1^ for interfacial peptides with solution pH of 3 (red) and 7 (black). The two spectra reflect difference in both nonresonance contribution and resonant bands, we further performed spectra fitting to deconvolute different bands, detailed fitting parameters were shown in Table S1. Fitting results reveal several bands from backbone and side chains of peptides, namely, the symmetric stretch of the COO^–^ group at ∼1467 cm^–1^,^19^ the amide II mode at ∼1540 cm^–1^,^20^ the asymmetric stretch of the COO^–^ group at ∼1597 cm^–1^,^21,22^ and the C=O mode of the COOH group at ∼1747 cm^–1^.^22^ In addition, the amide I vibrations, in the region of ∼1600–1700 cm^–1^ and arising mainly from the C=O vibration from the C(O)–N(H) bonds in the backbone, are particularly interesting for proteins and peptides. Since the frequency of amide I band is very sensitive to the backbone secondary structure, the resonance frequency has been widely used to differentiate folding structures such as random coil, α-helix, and β-strand.^17,23^ Fitting analysis (Table S1) reveals three amide I bands for Aβ_16–22_ peptides at pH 3: a positive band at 1640 cm^–1^ assigned to the B2 mode of β-strand, a small negative one at 1658 cm^–1^ assigned to the β-turn,^24,25^ and another positive peak at 1678 cm^–1^ assigned to the B1 mode of antiparallel β-strand.^26^ The amplitude of the high-frequency band at 1678 cm^–1^ is the largest among amide I peaks, suggesting the dominating β-strand secondary structure of interfacial peptides. The β-strand structure is further evidenced by the amide II signal at ∼1540 cm^–1^, which, according to a recent study,^20^ is only detectable for aggregated β-strand structures. By contrast, only β-turn bands were observed for the peptides at pH 7.

**Figure 2:**
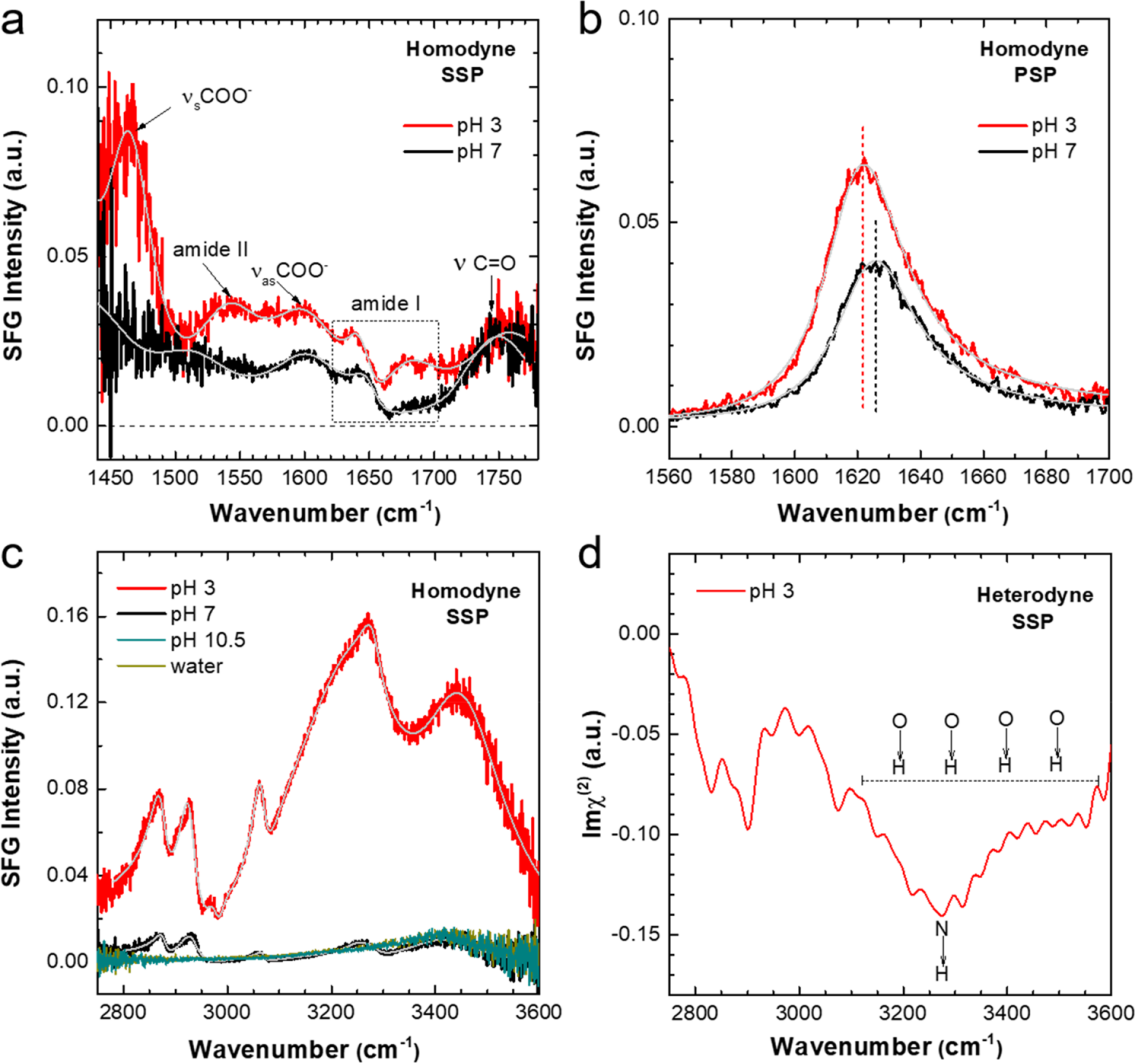
(a) Homodyne SSP SFG spectra in the frequency region 1440–1800 cm^–1^ for Aβ_16–22_ peptides at the air–water interface with bulk solution pH of 3 (red) and 7 (black). Characteristic bands from backbone and side chains are marked. (b) Homodyne PSP SFG spectra in amide I region for interfacial peptides at pH 3 (red) and 7 (black). (c) Homodyne SSP SFG spectra in CH/OH region for Aβ_16–22_ peptides at air–water interface with solution pH of 3 (red), 7 (black), and 10.5 (cyan). The spectrum for pure water (dark yellow) is included as reference, and is indistinguishable from the pH 10.5 spectrum. (d) Heterodyne SSP SFG spectra in CH/OH region for interfacial peptides at pH 3. In parts a–c, the spectra fits (gray) were superimposed into experimental homodyne spectra.

Figure 2b shows the amide I spectra under chiral PSP polarization combination. These chiral PSP spectra, suppressing the achiral side chain modes, provide a direct probe of backbone folding.^27,28^ It should be noted that the Aβ_16–22_ peptides in bulk solution are in centrosymmetric environment; therefore, the chiral SFG signals, as generated from peptides in dilute solutions, are assumed to be very small and are canceled out by each other. In this context, the detected chiral amide I signal originates mainly from interfacial peptides.^28^ The larger amplitude and lower resonance frequency (Table S2) for pH 3 spectra, again, point to the β-strand secondary structure for positively charged Aβ_16–22_ peptides,^21^ in contrary to the β-turn peptides at pH 7.

The folding structure of Aβ_16–22_ peptides at the air–water interface relies on the concerted (i) hydrophobic interaction exposed to air and (ii) hydrophilic interaction with water. To explore these interactions in more detail, we record SFG spectra in CH/OH region to probe the local hydrophobic sites and interfacial water simultaneously. Figure 2c shows the corresponding SSP spectra at different pH, the spectra for pure water is also included for comparison. The peptides are not surface active at basic pH (10.5), the spectrum is indistinguishable from that of water, which shows the broad OH response around 3100–3600 cm^–1^.^29,30^ In contrast, several CH bands can be identified in the spectra for neutral and acidic pH, assigned to vibrational modes of the hydrophobic side chains.^15,31−33^Table S3 presents detailed fitting parameters and band assignments. The most notable feature in Figure 2c is the intense OH bands for pH 3, which indicates that water molecules are aligned by the positively charged peptides.^30,34^ The absolute water orientation was determined by heterodyne-detected SFG (HD-SFG). In contrast to homodyne SFG, where the square of the second-order susceptibility |χ^(2)^|^2^ is measured, the HD-SFG allows the imaginary part of χ^(2)^ to be determined, and the sign of Im χ^(2)^ determines the net orientation of transition dipole moment of the probed vibrational modes, which informs about molecular orientation.^35^Figure 2d shows the Imχ^(2)^ spectra for the peptides at pH 3, the distinct negative OH bands indicates the downward oriented (toward bulk water) OH dipoles of water molecules. For charged peptide interfaces, the OH signal of water stems from both the interfacial layer (χ^(2)^-contribution) and the diffuse layer (χ^(3)^).^19,34,36^ For the 10 mM salt concentration in the present work, the diffuse layer contribution dominates, and as such, the SFG intensity mainly reports OH alignment of water molecules, those are within ∼3 nm below top surface and extending into bulk solution. Interestingly, the NH peak exclusively from peptide molecules (i.e., χ^(2)^-contribution) also appears as negative at 3270 cm^–1^, which implies that the NH dipoles also point downward.

To obtain a detailed molecular picture of interfacial peptides, we complement SFG results by metadynamics simulations. Metadynamics is a computational technique to enhance conformational sampling in standard molecular dynamics simulations. The conformational sampling is accelerated by a history-dependent bias potential along few selected degrees of freedom of the system, which, often referred as collective variables (CVs), were exploited to reconstruct the free-energy profile for complex biomolecules at different interface.^37−39^ Metadynamics simulations were conducted in 430 ns on a single positively charged peptide and on a single zwitterionic Aβ_16–22_ peptide, corresponding to the pH of 3 and 7, respectively, each placed at water/vacuum interface (see the section “Simulation description” in the Supporting Information). The dihedral angle between the phenyl rings within Aβ_16–22_ peptide, defined by the (C_α_–C_β_)–(C_α_–C_β_) atoms of PHE_19_ and PHE_20_, respectively (see Figure S4), was exploited as the collective variable ensuring adequate exploration of the peptide conformational space.^8^ Each system consists of a 60 × 60 × 100 Å^3^ “slab” of water embedded in a simulation box of 60 × 60 × 360 Å^3^, which guarantee vacuum along the *z* direction, and a proper number of counterions were used to guarantee neutrality and commensurate ionic strength between systems. (Figure S2)

In agreement with the SFG data, both the positively charged and zwitterionic peptides remain at water/vacuum interface for the entire duration of the simulation (Figure S3). Parts a and b of Figure 3 illustrate the typical snapshots for zwitterionic and positively charged peptides, respectively. In agreement with our SFG results, the charged peptides fold into elongated β-strand conformation, as in contrast to the less folded, bent-over motif for zwitterionic ones. Figure 3c presents the free energy profiles according to the dihedral angles of phenyl rings. For both charged and zwitterionic peptides, the *cis-oid* is the preferable conformation with lower free energy. Yet, the free energy barriers between *cis-oid* and *trans-oid* is much lower for the charged peptides as compared to zwitterionic form (6 vs 15 kJ/mol), which implies efficient interconversion between two conformational states for the charged state. The different local conformation flexibility for two types of peptides hints on their different backbone conformation. The ensemble backbone structures for two types of peptides were quantitatively juxtaposed with a stable β-sheet polymorph I.^40^Figure 3d presents the root-mean-square deviation (RMSD) with respect to the standard β-strand conformation.^8^ The RMSD values for zwitterionic peptides have rather broad distribution toward higher values. By contrast, the RMSD values for charged peptides populate at smaller values with narrower distribution, which supports their dominating β-strand secondary structure.

**Figure 3:**
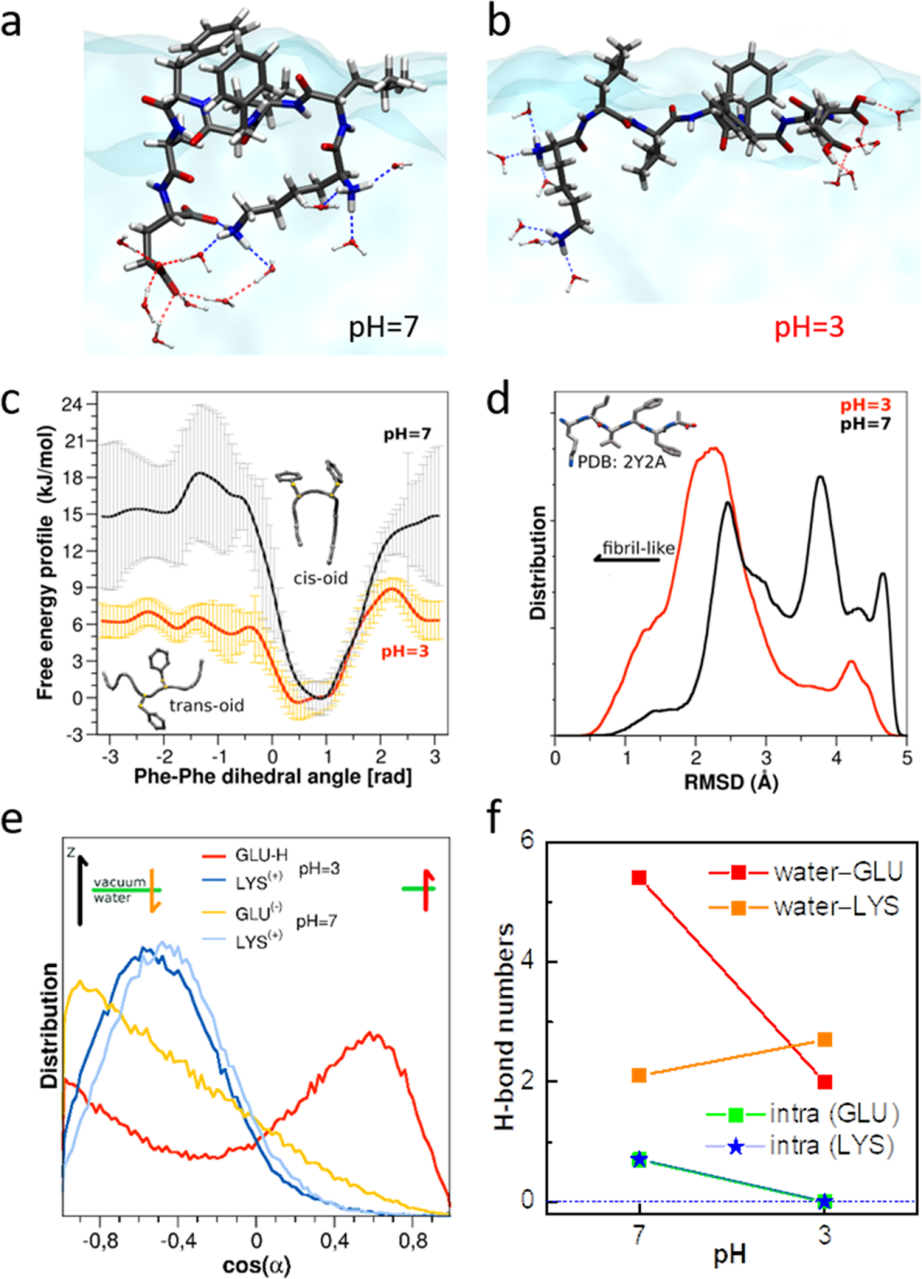
(a, b) Typical snapshots of zwitterionic (pH 7, a) and positively charged (pH 3, b) Aβ_16–22_ peptides at the water–vacuum interface: peptides differ in the protonation states of the carboxylic groups, at pH 7 being deprotonated and negatively charged while at pH 3 being protonated and neutral. (c) Free energy profiles according to the dihedral angles, as defined by the two phenyl rings within the positively charged (pH 3) and zwitterionic (pH 7) peptides. (d) Distribution of root-mean-square deviation (RMSD) with respect to the stable β-sheet polymorph (PDB code: 2y2a) for positively charged (pH 3) and zwitterionic (pH 7) peptides at the water–vacuum interface. (e) Orientation distribution of lysine (LYS) and glutamic acid (GLU) side chains for positively charged (pH 3) and zwitterionic (pH 7) peptides at the air–vacuum interface. (f) Average numbers of hydrogen bonds for side chain groups in positively charged (pH 3) and zwitterionic (pH 7) peptides at the air–vacuum interface. The LYS and GLU charge groups can form: (i) inter-hydrogen bonds with water molecules and (ii) intra- hydrogen bonds within Aβ_16–22_ peptides.

Now the question arises: What drives the refolding of positively charged peptides into defined β-strand conformation? The main difference for charged peptides compared to zwitterionic form is the protonated glutamic acid side chains. We first analyze the orientation distribution for lysine (LYS) and glutamic acid (GLU) side chains, as shown in Figure 3e. The orientational distributions of all side chains are shown in Figure S5. The angle α is defined as the bond, between Cα and center of mass of side chain, with respect to the surface normal (*z*-axis). The LYS side chains orient similarly for two peptide forms. In contrast, the GLU side chains show drastically different orientation distribution: the GLU side chains of zwitterionic peptides orient into bulk water with a broad angular distribution. In contrast, the GLU side chains of charged peptides orient oppositely, pointing toward the air, with a narrower angle distribution.

The opposite orientations for GLU side chains are closely related to the interfacial hydrogen bonding network. We calculate the average numbers of hydrogen bonds (H-bonds) per time frame between side-chain (GLU or LYS) groups and water molecules in the first hydration shell (Figure 3f). Compared with zwitterionic peptides, the hydrogen bond number for NH^3+^ groups from LYS side chains only increase slightly for charged peptides. In stark contrast, the number for COO^–^/COOH groups from GLU side chains decreases substantially, from 5.5 to 2.0, accompanying the protonation of the COO^–^ group. We further calculate the numbers of intramolecular H-bonds within the peptides. The charging groups of LYS and GLU side chains can both form intra- H-bonds within the zwitterionic peptides, which also explains their bent motif (Figure 3 a). By contrast, these intra-H-bonds are inhibited for positively charged peptides. Therefore, the H-bonds analysis shows that both inter- and intra-H-bonds are suppressed for positively charged peptides, and the reduced free energy gain is not adequate to solvate peptides, repelling them toward the hydrophobic air side (Figure S6). The interfacial accumulation of charged peptides, as caused by the decreasing solubility, is also proven by our surface tension measurement (Figure S1). The repelled charged peptides refold into an extended β-strand conformation; within this conformation, the increasing hydrophobic contacts are assumed to promote their interfacial assembly into elongated flakes macroscopically.

In conclusion, we have combined SFM, SFG spectroscopy, and enhanced sampling atomistic simulations to probe the Aβ_16–22_ peptides at the air–water interface with different solution pH values. At neutral pH, the zwitterionic peptides adopt bending conformation by forming intra-hydrogen bonds. At acidic pH, the protonated COOH groups in glutamic acid side-chain reduce both inter-H-bonds with water and intra-H-bonds within peptide molecules. The modification of the interfacial H-bonds drives the refolding of peptides into extended β-strand conformation, which further promotes their macroscopic assembly.

Despite the complexity in interfacial assembly of Aβ peptides, our results provide molecular-level details on how the fiber conformation transition and macroscopic assembly can be triggered by modulating ambient pH. Our insights may stimulate new strategies to control peptide aggregation for future amyloidosis therapeutics.

## Supporting Information Available

More characterization details on SFM, surface pressure, and SFG; fitting results of SFG spectra; and additional simulation details and data (PDF)
