# Supplementary material for: Acidic pH Promotes Refolding and Macroscopic Assembly of Amyloid β (16–22) Peptides at the Air–Water Interface
Source: J Phys Chem Lett. 2022 Jul 15;13(29):6674–9. doi: 10.1021/acs.jpclett.2c01171 (PMC9340808; doi:10.1021/acs.jpclett.2c01171)
Supplement: Supplementary file 1 — jz2c01171_si_001.pdf [file jz2c01171_si_001.pdf]

Supporting Information

Acidic pH Promotes Refolding and Macroscopic Assembly of Amyloid β (16-22) Peptides at the Air-Water Interface

Hao Lu,[1] Luca Bellucci,[2] Shumei Sun,[3] Daizong Qi,[1] Marta Rosa,[4,5] Rüdiger Berger,[1] Stefano Corni,[4,5] Mischa Bonn,[1*]*

[1] Max Planck Institute for Polymer Research, Ackermannweg 10, 55128, Germany

E-mail: lu@mpip-mainz.mpg.de; bonn@mpip-mainz.mpg.de

[2] NEST – Istituto di Nanoscienze del Consiglio Nazionale delle Ricerche CNR-NANO and Scuola Normale Superiore, Piazza S. Silvestro 12, Pisa, 56127, Italy

[3] Department of Physics and Applied Optics Beijing Area Major Laboratory, Beijing Normal University, Beijing, 100875, China

[4] Istituto di Nanoscienze del Consiglio Nazionale delle Ricerche CNR-NANO, 41125 Modena, Italy

[5] Dipartimento di Scienze Chimiche, Universita` di Padova, 35131 Padova, Italy

Table of Content:

Scanning Force Microscopy (SFM) S2

Surface Pressure measurements S3

Vibrational Sum Frequency Generation (SFG) spectroscopy S4 – S9

Simulation description: S10-S15

References S16

Scanning Force Microscopy (SFM):

Sample preparation: Atomically flat mica substrates were placed inside a home built Teflon trough. $A\beta_{16–22}$ peptide solution in 5 mL volume at pH 3 and 7 were injected into trough, the peptide solutions were equilibrated for 2 hours, which ensures the fully adsorption and assembly of peptides at the air-water interface. Bulk peptide solution (below interfacial peptides) was removed slowly and carefully using a syringe, which was connected to the pinhole at the bottom of the trough. The bottom plane of the Teflon trough is tilted with respect the horizontal water surface, this geometry allows the removal of bulk peptides, while results in the deposition of interfacial peptides onto mica surface. The deposited peptide layers on mica were subsequently measured by SFM.

SFM measurements were performed with a commercial instrument (Bruker Dimension ICON) operated in tapping mode (OTESPA, with a nominal resonance frequency of 300 kHz and a spring constant of 26 N/m) in air.

Surface Pressure measurements

Surface pressure has been measured using a Langmuir tensiometer (Kibron, Finland). The Teflon trough was thoroughly cleaned sequentially with acetone, ethanol, and milliQ water, and dried under a nitrogen stream prior to measurements. The surface pressure (π) was normalized with pure water to 0 mN/m.

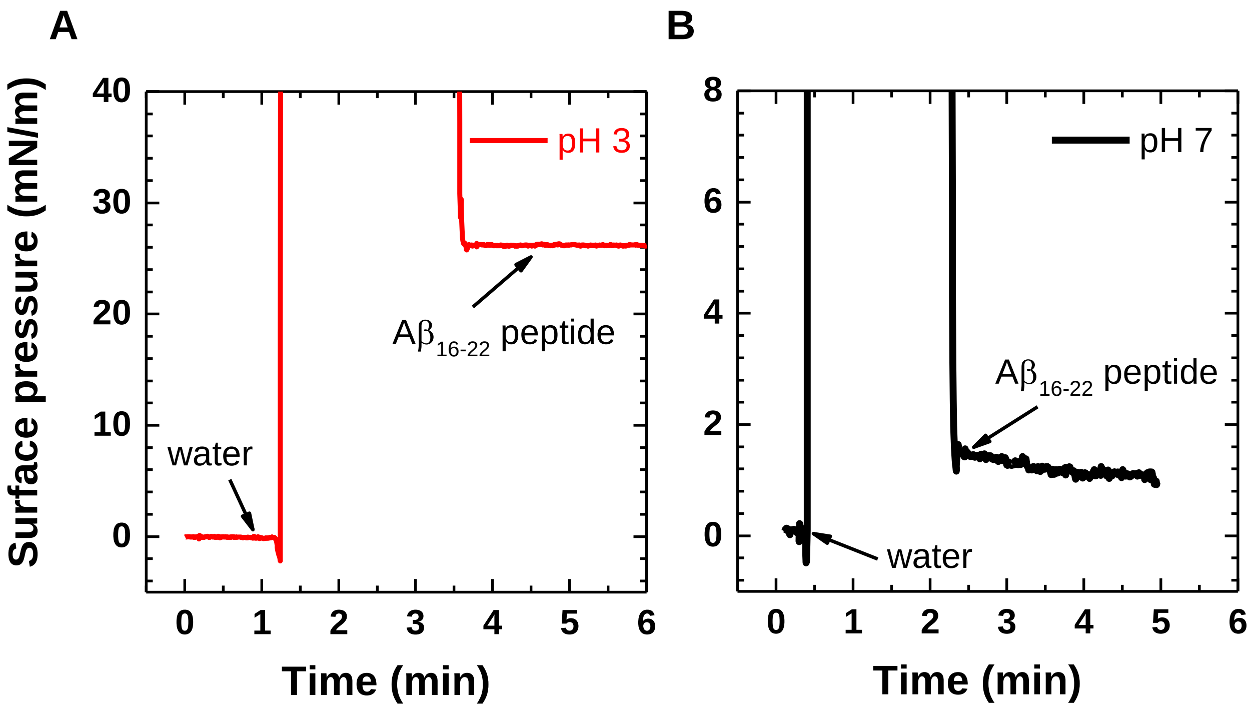

Figure S1: **(A-B)** surface pressure of A$\beta_{16\text{–}22}$ peptides at air-water interface with solution pH of 3 (A) and 7 (B). More A$\beta_{16\text{–}22}$ peptide molecules absorb at the water surface of acidic solution, reaching a higher surface pressure value of 26 mN/m.

Vibrational Sum Frequency Generation (SFG) spectroscopy

Homodyne SFG: The vibrational SFG spectra were obtained by overlapping, in time and space, the visible and IR pulses. A Ti:Sapphire amplified system (Spitfire Ace, Spectra Physics Inc.) delivers 35 fs long pulses at a central wavelength of ~800 nm and 1 KHz repetition rate. The beam is split in two parts: one it is spectrally narrowed using a Fabry-Perot etalon to achieve spectral resolution of 15 cm^{-1} (lambda=800 nm, E~25 mJ/pulse). The other part is used to generate tunable broadband IR pulses thanks to a parametric optical amplifier followed by a noncollinear difference frequency generation module (TOPAS Prime). The average power is 2 μJ/pulse at a wavelength of 6000 nm and 3 μJ/pulse at a wavelength of 3000 nm. Visible and IR beams are focused onto the sample using respectively a 20 cm and 5 cm focal length (FL) lenses. The polarization of both beams can be controlled (S or P) with a polarizer and a half waveplate. Beams are temporally and spatially overlapped at the sample position. The SFG signal is generated with Visible and IR beam angles of 55° and 60° respective to the surface normal, and the signal is collimated using a 20 cm FL lens, and focused into a spectrograph using a 5 cm FL achromatic lens, dispersed by a grating and collected by an Electron-multiplying CCD (EMCCD) camera. The polarization of the SFG signal can be well controlled like Visible and IR beams.

Each SFG spectrum was acquired for 10 minutes, and the spectra are normalized by non-resonance reference spectra of z-cut quartz crystal after background correction. Spectra were recorded in the SSP (sum, visible, and infrared) or PSP polarization combination. For SFG experiments in amide I region, D_2O solvent was used to avoid the spectra interference from the bending mode of H_2O, and spectra were calibrated by the absorption bands of water vapor. Spectra recorded in CH/OH region were referenced by the absorption bands of polystyrene.

SFG spectra were fitted by Lorentzian peak shapes according to the following equation:

$$I_{SFG} \propto \left|\chi^{(2)}\right|^2 = \left|\chi_{NR}^{(2)} + \chi_R^{(2)}\right|^2 = \left|A_{NR}e^{i\Phi_{NR}} + \sum \frac{A_n}{\omega_{IR} - \omega_n - i\Gamma_n}\right|^2 \tag{1}$$

In equation (1) above, the susceptibility $\chi^{(2)}$ consists of a non-resonant ($\chi_{NR}^{(2)}$) and a resonant ($\chi_R^{(2)}$) term. A_{NR} and ϕ_{NR} are the amplitude and phase of non-resonant signal, respectively. A_n is the amplitude of resonant signal, ω_n is the resonant frequency, ω_{IR} is the infrared frequency, and Γ_n is the width of transition.

Heterodyne SFG: In heterodyne detection, SFG signals are generated from both local oscillator (LO) and from the sample. The two SFG signals are delayed in time with respect to each other by passing the LO SFG beam through a silica plate. The two SFG beams are sent into a monochromator and detected by EMCCD. The interference pattern of the two SFG signals are analyzed using a Fourier transformation. The spectra in time domain was processed using rectangular function, finally both the real and imaginary parts of $\chi^{(2)}$ ($\mathrm{Im}\chi^{(2)}$) can be extracted, by referencing the heterodyne SFG signal of the sample with that for z-cut quartz whose SFG phase is already known.

Table S1. Peak fitting parameters and assignment[a] for SSP SFG spectra in Figure 2a, for the A$\beta_{16\text{-}22}$ peptides at water surface with solution pH of 3 and 7.

	pH 3		pH 7
$\mathrm{Im}\chi^{(2)}_{NR}$	0.00158	$\mathrm{Im}\chi^{(2)}_{NR}$	-0.02116
$\mathrm{Re}\chi^{(2)}_{NR}$	0.0116	$\mathrm{Re}\chi^{(2)}_{NR}$	0.07304
A_1	4.7	A_1	4.7
ω_1 (cm^{-1}) ν_s (COO$^-$)+δ(CH$_2$)	1419	ω_1 (cm^{-1}) ν_s (COO$^-$)+δ(CH$_2$)	1404
Γ_1 (cm^{-1})	23	Γ_1 (cm^{-1})	28
A_2	5.3	A_2	3.5
ω_2 (cm^{-1}) ν_s (COO$^-$) + ν_{sc} (CH$_2$)	1467	ω_2 (cm^{-1}) ν_s (COO$^-$) + ν_{sc} (CH$_2$)	1450
Γ_2 (cm^{-1})	25	Γ_2 (cm^{-1})	40
A_3	4.3	A_3	4.7
ω_3 (cm^{-1}) amide II	1540	ω_3 (cm^{-1}) amide II	1521
Γ_3 (cm^{-1})	38	Γ_3 (cm^{-1})	50
A_4	4.5	A_4	4.2
ω_4 (cm^{-1}) ν_{as} (COO$^-$)	1597	ω_4 (cm^{-1}) ν_{as} (COO$^-$)	1607
Γ_4 (cm^{-1})	40	Γ_4 (cm^{-1})	39
A_5	0.6	A_5	1.4
ω_5 (cm^{-1}) B2 β- strand	1640	ω_5 (cm^{-1}) β turn	1650
Γ_5 (cm^{-1})	13	Γ_5 (cm^{-1})	17
A_6	-0.5	A_6	-0.6
ω_6 (cm^{-1}) β- turn	1658	ω_6 (cm^{-1}) β turn	1658
Γ_6 (cm^{-1})	13	Γ_6 (cm^{-1})	14
A_7	1.77	A_7	6.6
ω_7 (cm^{-1}) B1 anti-parallel β- strand	1678	ω_7 (cm^{-1}) C=O	1750
Γ_7 (cm^{-1})	29	Γ_7 (cm^{-1})	39
A_8	4.8		

ω_8 (cm^{-1}) C=O	1747
Γ_8 (cm^{-1})	44

^{a} ν_s – symmetric stretching, ν_{as} – asymmetric stretching, δ – bending motion, ν_{sc} –scissor

Table S2. Peak fitting parameters and assignment for PSP SFG spectra in Figure 2b, for the Aβ_{16-22} peptides at water surface with solution pH of 3 and 7.

	pH 3		pH 7
$\mathrm{Im}\chi^{(2)}_{NR}$	0.03682	$\mathrm{Im}\chi^{(2)}_{NR}$	0.0359
$\mathrm{Re}\chi^{(2)}_{NR}$	-0.03618	$\mathrm{Re}\chi^{(2)}_{NR}$	-0.02429
A	3.3	A	2.6
ω (cm^{-1})<br>B mode of β- strand	1620	ω (cm^{-1})<br>β- turn (long range order)	1624
Γ (cm^{-1})	16	Γ (cm^{-1})	16

Table S3. Peak fitting parameters and assignment for CH/OH SFG spectra in Figure 2c, for the A$\beta_{16\text{-}22}$ peptides at water surface with solution pH of 3 and 7.

	pH 3		pH 7
$Im\chi^{(2)}_{NR}$	-0.02727	$Im\chi^{(2)}_{NR}$	0.01061
$Re\chi^{(2)}_{NR}$	-0.0519	$Re\chi^{(2)}_{NR}$	-0.02721
A_1	-1.7	A_1	-0.7
ω_1 (cm^{-1}) ν_s CH_3	2875	ω_1 (cm^{-1}) ν_s CH_3	2877
Γ_1 (cm^{-1})	18	Γ_1 (cm^{-1})	13
A_2	-2.6	A_2	-2.1
ω_2 (cm^{-1}) ν_{FR} CH_3	2930	ω_2 (cm^{-1}) ν_{FR} CH_3	2934
Γ_2 (cm^{-1})	20	Γ_2 (cm^{-1})	21
A_3	-0.2	A_3	-0.4
ω_3 (cm^{-1}) ν_s CH_2 (from side chain)	2974	ω_3 (cm^{-1}) ν_2 (phenyl)	3068
Γ_3 (cm^{-1})	11	Γ_3 (cm^{-1})	10
A_4	0.2	A_4	-0.4
ω_4 (cm^{-1}) ν_{as} CH_3	2989	ω_4 (cm^{-1}) ν_{OH}	3256
Γ_4 (cm^{-1})	11	Γ_4 (cm^{-1})	22
A_5	2.8	A_5	-0.8
ω_5 (cm^{-1}) ν_{20A} (phenyl)	3048	ω_5 (cm^{-1}) ν_{NH}	3280
Γ_5 (cm^{-1})	56	Γ_5 (cm^{-1})	23
A_6	-1.1	A_6	-9.6
ω_6 (cm^{-1}) ν_2 (phenyl)	3067	ω_6 (cm^{-1}) ν_{OH}	3427
Γ_6 (cm^{-1})	14	Γ_6 (cm^{-1})	89
A_7	-33.8		
ω_7 (cm^{-1}) ν_{OH}	3254		
Γ_7 (cm^{-1})	133		
A_8	-1.0		

ω_8 (cm^{-1}) ν_{NH}	3279
Γ_8 (cm^{-1})	25
A_9	-24.5
ω_9 (cm^{-1}) ν_{OH}	3447
Γ_9 (cm^{-1})	105

Simulation description:

Molecular dynamics simulations were carried out with GROMACS[7](v2018.2) patched with PLUMED [(3, (10] (v2.5.0). The systems was modelled with gromacs tools and VMD[(8]. The $A\beta_{16-22}$ segment (i.e. residues KLVFFAE) was extracted from the first NMR structure deposited in the protein data bank (PDB code 1IYT). Both N- and C-termini were uncapped. Two different protonation states of $A\beta_{16-22}$ were taken in account for acidic (pH 3) and neutral (pH 7) conditions, rendering the peptides into positively charged and zwitterionic form, respectively. Protonation state for lysine and glutammic acid residue at different pH was established after the evaluation of the pKa constant with PROPKA tool. [(11]. $A\beta_{16-22}$ peptides were placed at the water/vacuum interfaces of a water slab of 60x60x100 A^3 (see Figure S); 6 Cl^- and 2 Na^+ ions were added to pH 3, whereas 2 Cl^- and 2 Na^+ were added to pH 7. $A\beta_{16-22}$ was treated with the classical force field OPLS [(9], while the SPC/E model[(2] was used for water molecules, as it was shown that this model was reliable for describing the water/vacuum interface with sufficient accuracy [(11].

The simulations were conducted using periodic boundary conditions (PBC) in all the three dimensions; nevertheless, the long-range part of the electrostatic potential were treated with Particle-Mesh-Ewald (PME)[5] taking in account the periodicity only along x and y directions by exploiting the pseudo-2d Ewald summation (3DC)[(12]. The system had a slab geometry and the dimension of x-y plane was the same of the water box. The z-dimension, accordingly to the reduced Ewald-geometry[(12], was set three times larger than the height of the water slab. The final simulation box was 60x60x360 $Å^3$ for both systems. The Fourier spacing for the PME summation was set 1.2 Å whereas the distance cut off for non-bonded interactions was set to 13 Å. All MD simulations were performed in the NVT ensemble (T=300 K) controlled via the stochastic velocity-

rescaling thermostat.[(4] Integration time step was set to 2 fs and all bonds were treated as holonomic constraints using the LINCS algorithm[6].

Both systems were minimised and subjected to 2 ns of MD simulations. The final conformations were used to perform 430 ns of well tempered metadynamics[1] exploiting as a collective variable the dihedral angle defined by (CB-CA)–(CA-CB) atoms of the adjacent phenyl-alanine residues (Figure S). The width of the Gaussian function was 0.2 rad and the initial height of the Gaussian functions was 1.5 kJ/mol. The “biasfactor” for well-tempered metadynamics was set to 15. The bias-potential was regularly updated at every 4 ps intervals throughout the simulations.

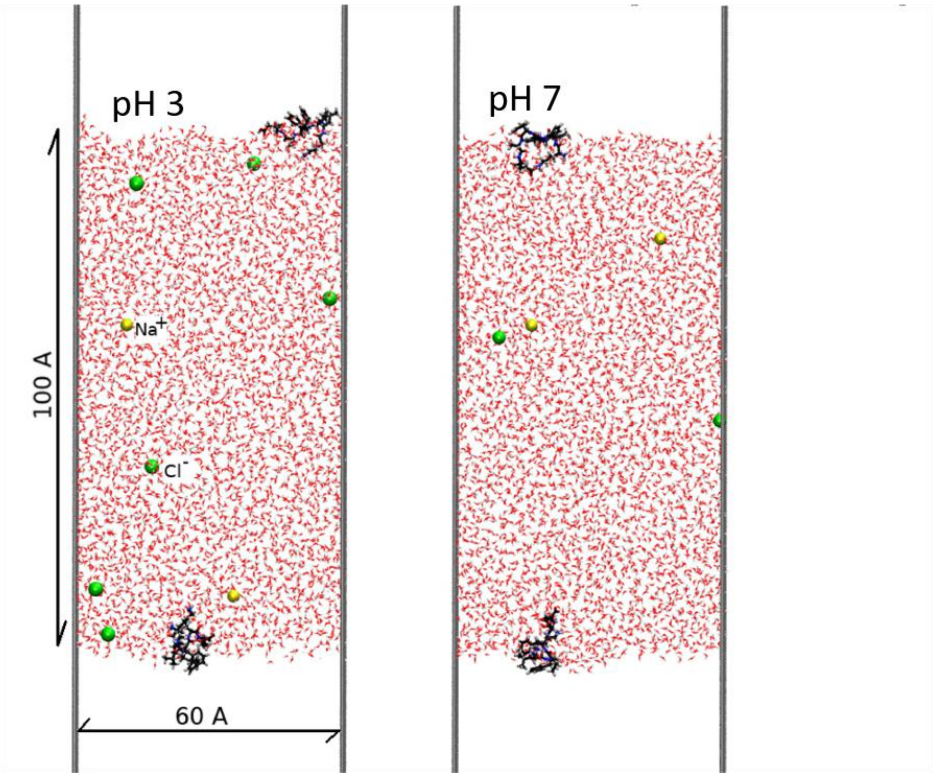

Figure S2: Simulation system composed of water molecules, $A\beta_{16–22}$ peptides at water/vacuum interface, and counter ions. Left: positively charged $A\beta_{16–22}$ corresponding to pH 3; Right: zwitterionic $A\beta_{16–22}$ corresponding to pH 7. Na^+ and Cl^- are in yellow and green, respectively.

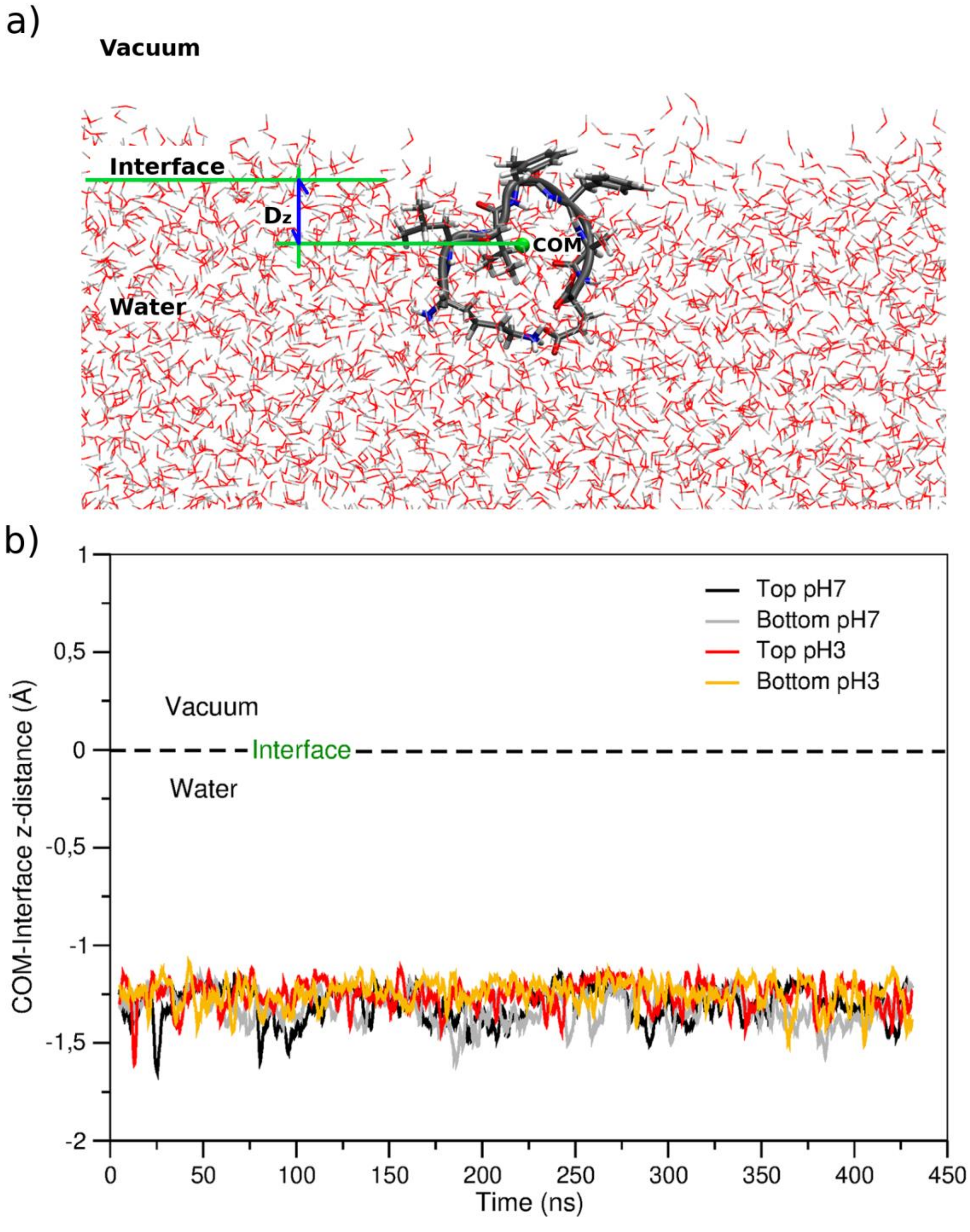

Figure S3: **(a)** Illustration of $A\beta_{16-22}$ peptides at water/vacuum interface in the simulation box. **(b)** Location of $A\beta_{16-22}$ peptides at two pH over 400 ns simulation runs. The z-distance is the distance defined for the center of mass (COM) of peptides with respect to the interface.

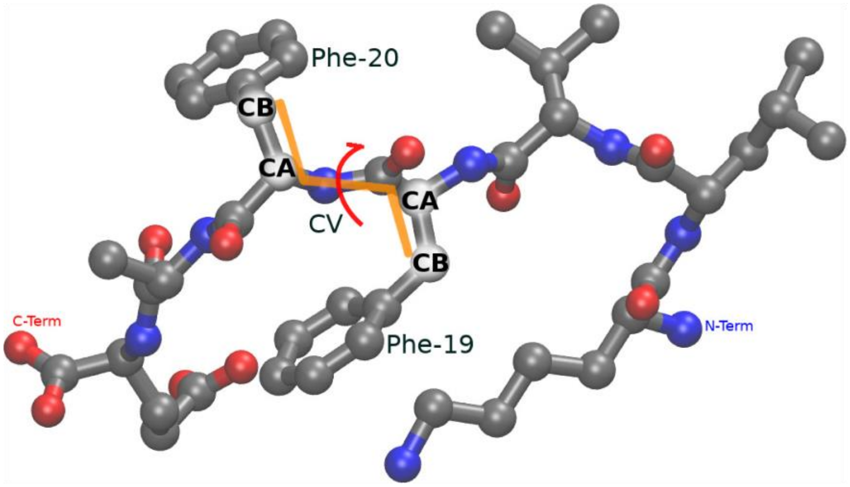

Figure S4: Definition of the collective variable (CV) used in metadynamics simulations to explore the peptide conformations at water/vacuum interface and to reconstruct the free energy profile in function of the relative orientation of the phenyl rings.

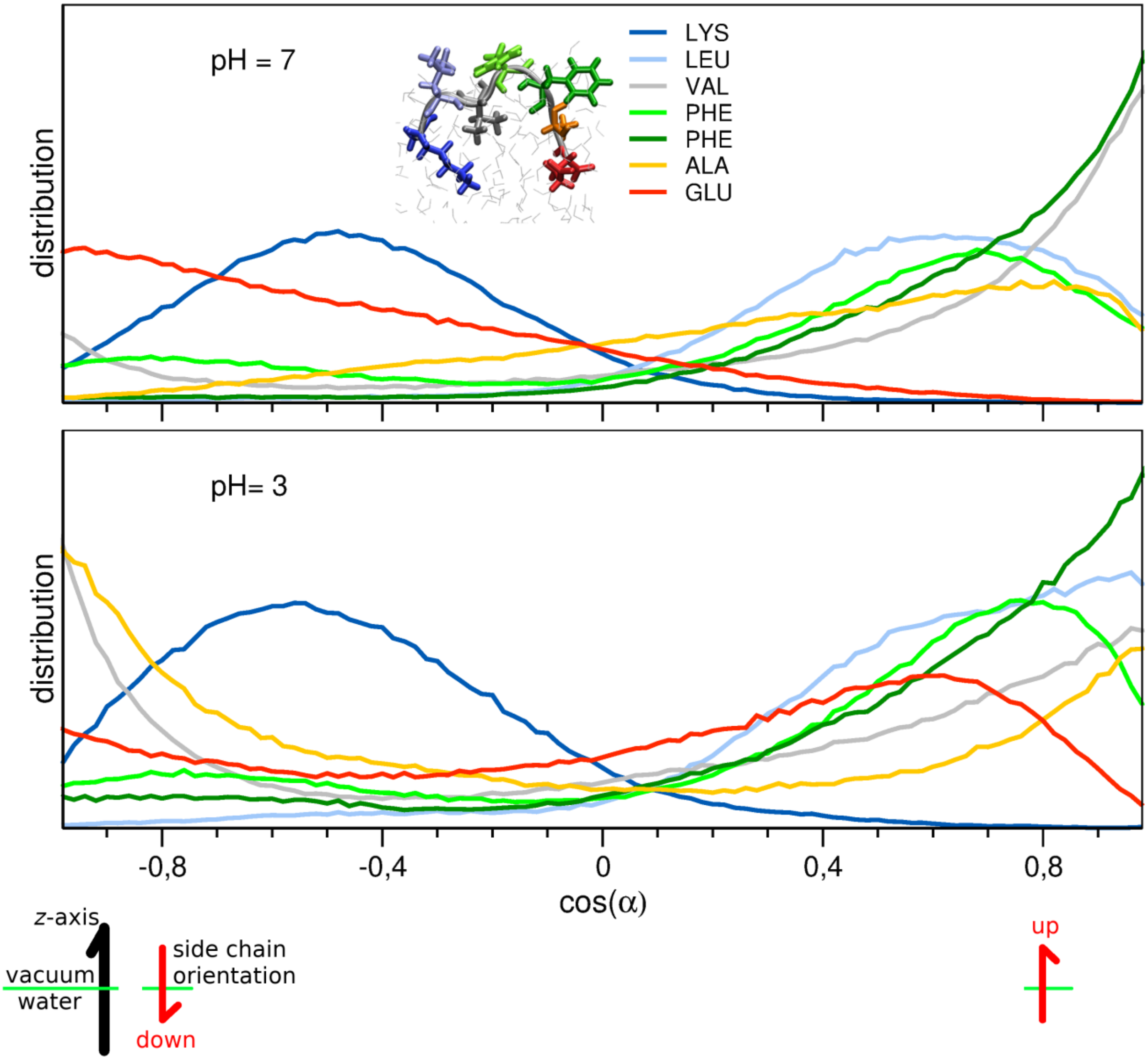

Figure S5: Orientation distribution of different side chains for zwitterionic (Up, pH 7) and positively charged (Down, pH 3) A$\beta_{16\text{–}22}$ peptides at air-vacuum interface. The orientations of hydrophobic side chains agree well with that revealed from the heterodyne SFG spectra in Figure 2 (d).

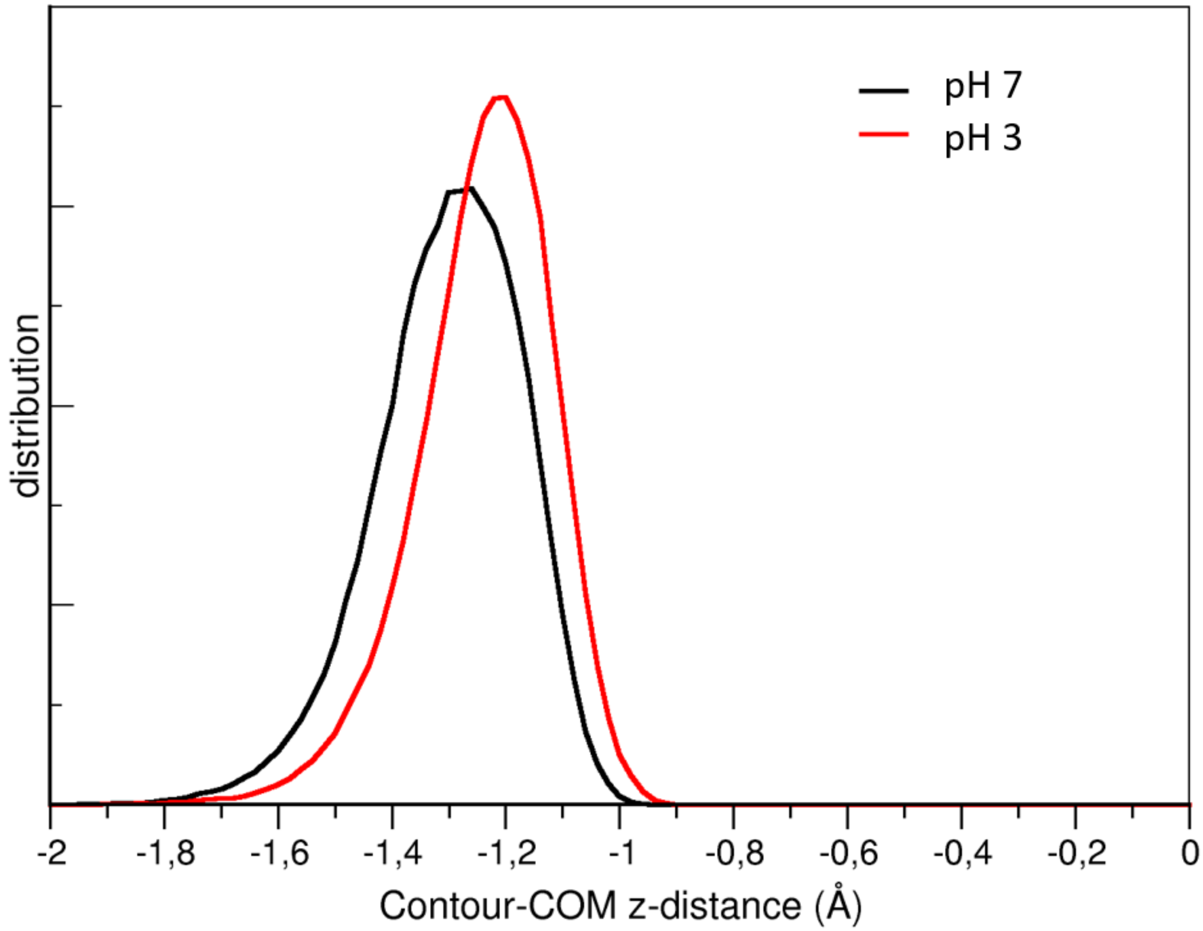

Figure S6: Distribution of the location (z) of $A\beta_{16–22}$ peptides at two pH. The location z-distance is defined for the center of mass (COM) of peptides with respect to the interface (see Figure S3 (a)).

References

(1) A. Barducci, G. Bussi, and M. Parrinello. well-tempered metadynamics: a smoothly converging and tunable free-energy method. *Phys. Rev. Lett.* **2008**, *100* (2), 20603.

(2) HJC Berendsen, JPM Postma, WF Van Gunsteren, and J. Hermans. interaction models for water in relation to protein hydration. *Intermolecular forces* **1981**, *11* (1), 331–342.

(3) M. Bonomi, D. Branduardi, G. Bussi, C. Camilloni, D. Provasi, P. Raiteri, D. Donadio, F. Marinelli, F. Pietrucci, R.A. Broglia, and M. Parrinello. Plumed: a portable plugin for free-energy calculations with molecular dynamics. *Comp. Phys. Commun.*, **2009**, *180* (10), 1961–1972.

(4) Giovanni Bussi, Davide Donadio, and Michele Parrinello. canonical sampling through velocity rescaling. *J. Chem. Phys.* **2007**, *126* (1), 014101.

(5) U. Essmann, L. Perera, M. L. Berkowitz, T. Darden, H. Lee, and L. G. Pedersen. a smooth particle mesh Ewald method. *J. Chem. Phys.* **1995**, *103* (19), 8577–8593.

(6) B. Hess. P-lincs: a parallel linear constraint solver for molecular simulation. *J. Chem. Theory Comput.* **2008**, *4* (1), 116–122.

(7) B. Hess, C. Kutzner, D. Van Der Spoel, and E. Lindahl. gromacs 4: algorithms for highly efficient, load-balanced, and scalable molecular simulation. *J. Chem. Theory Comput.* **2008**, *4* (3), 435–447.

(8) W. Humphrey, A. Dalke, and K. Schulten. VMD: visual molecular dynamics. *J. Mol. Graph.* **1996**, *14* (1), 33–38.

(9) William L. Jorgensen, David S. Maxwell, and Julian Tirado-Rives. development and testing of the opls all-atom force field on conformational energetics and properties of organic liquids. *J. Am. Chem. Soc.* **1996**, *118* (45), 11225–11236.

(10) Gareth A Tribello, Massimiliano Bonomi, Davide Branduardi, Carlo Camilloni, and Giovanni Bussi. plumed 2: new feathers for an old bird. *Comput. Phys. Commun.* **2014**, *185* (2), 604–613.

(11) C Vega and E De Miguel. surface tension of the most popular models of water by using the test-area simulation method. *J. Chem. Phys.* **2007**, *126* (15), 154707.

(12) In-Chul Yeh and Max L Berkowitz. Ewald summation for systems with slab geometry. *J. Chem. Phys.* **1996**, *111* (7), 3155–3162.

(13) Hui Li, Andrew D. Robertson, Jan H. Jensen. very fast empirical prediction and rationalization of protein pKa values. *Proteins: Struct. Funct. Genet.* **2005**, *61*, 704-721.
